# Unrecognized hypertension among a general adult Ghanaian population: An urban community-based cross-sectional study of prevalence and putative risk factors of lifestyle and obesity indices

**DOI:** 10.1371/journal.pgph.0001973

**Published:** 2023-05-24

**Authors:** Enoch Odame Anto, Wina Ivy Ofori Boadu, Emmanuel Ekow Korsah, Ezekiel Ansah, Eric Adua, Joseph Frimpong, Patience Nyarkoa, Valentine Christian Kodzo Tsatsu Tamakloe, Emmanuel Acheampong, Evans Adu Asamoah, Stephen Opoku, Ebenezer Afrifa-Yamoah, Max Efui Annani-Akollor, Christian Obirikorang

**Affiliations:** 1 Faculty of Allied Health Sciences, Department of Medical Diagnostics, College of Health Sciences, Kwame Nkrumah University of Science and Technology, Kumasi, Ghana; 2 School of Medical and Health Sciences, Edith Cowan University, Joondalup Drive, Perth, Australia; 3 Centre for Precision Health, ECU Strategic Research Centre, Edith Cowan University, Perth, Australia; 4 Rural Clinical School, Medicine and Health, University of New South Wales, Sydney, NSW Australia; 5 Department of Physiology, School of Medicine and Dentistry, College of Health Science, Kwame Nkrumah University of Science and Technology, Kumasi, Ghana; 6 Department of Molecular Medicine, School of Medicine and Dentistry, College of Health Science, Kwame Nkrumah University of Science and Technology, Kumasi, Ghana; 7 School of Science, Edith Cowan University, Joondalup, Australia; Universiti Malaya, MALAYSIA

## Abstract

Hypertension (HTN) is the leading cause of cardiovascular diseases. Nevertheless, most individuals in developing countries are unaware of their blood pressure status. We determined the prevalence of unrecognized hypertension and its association with lifestyle factors and new obesity indices among the adult population. This community-based study was conducted among 1288 apparently healthy adults aged 18–80 years in the Ablekuma North Municipality, Ghana. Sociodemographic, lifestyle characteristics, blood pressure and anthropometric indices were obtained. The prevalence of unrecognized HTN was 18.4% (237 / 1288). The age groups 45–54 years [aOR = 2.29, 95% CI (1.33–3.95), p = 0.003] and 55–79 years [aOR = 3.25, 95% CI (1.61–6.54), p = 0.001], being divorced [aOR = 3.02 95% CI (1.33–6.90), p = 0.008], weekly [aOR = 4.10, 95% CI (1.77–9.51), p = 0.001] and daily alcohol intake [aOR = 5.62, 95% CI (1.26–12.236), p = 0.028] and no exercise or at most once a week [aOR = 2.25, 95% CI (1.56–3.66), p = 0.001] were independently associated with HTN. Among males, the fourth quartile (Q4) of both body roundness index (BRI) and waist to height ratio (WHtR) [aOR = 5.19, 95% CI (1.05–25.50), p = 0.043] were independent determinants of unrecognized HTN. Among females, the third quartile (Q3) [aOR = 7.96, 95% CI (1.51–42.52), p = 0.015] and Q4 [aOR = 9.87 95% CI (1.92–53.31), p = 0.007] of abdominal volume index (AVI), the Q3 of both BRI and WHtR [aOR = 6.07, 95% CI (1.05–34.94), p = 0.044] and Q4 of both BRI and WHtR [aOR = 9.76, 95% CI (1.74–54.96), p = 0.010] were independent risk factors of HTN. Overall, BRI (AUC = 0.724) and WHtR (AUC = 0.724) for males and AVI (AUC = 0.728), WHtR (AUC = 0.703) and BRI (AUC = 0.703) for females yielded a better discriminatory power for predicting unrecognized HTN. Unrecognized hypertension is common among the apparently healthy adults. Increased awareness of its risk factors, screening, and promoting lifestyle modification is needed to prevent the onset of hypertension.

## Introduction

Chronic non-communicable diseases (CNCDs) are responsible for the majority of deaths worldwide. CNCDs cause an estimated 41 million deaths annually and accounts for 71% of all deaths globally [1,2]. This has placed a great burden on healthcare systems [3]. Hypertension (HTN) is a common widespread non-communicable disease and a major global public health concern [4]. Hypertension is a multifactorial clinical disorder depicted by high and sustained levels of blood pressure [5] and the commonest risk factor for cardiovascular diseases such as stroke and ischemic heart disease [6]. Despite HTN being a global health concern, many people with hypertension remain unrecognized/undiagnosed thus increasing the risk of morbidity and mortality [7].

HTN accounted for 10.8 million deaths in 2019 globally [4]. Findings from several studies show an increasing prevalence of HTN in low- and middle-income countries [8,9]. Currently, the economies of many developing nations are expanding at enormous rates. Unfortunately, this growth usually comes with the adoption of unhealthy lifestyles that outpaces advancements in healthcare [10]. Physical inactivity, alcohol intake and consumption of more westernized high-calorie diets impose a major burden on developing regions, where public awareness and knowledge of the harm they pose to health is limited [11]. In Africa, the estimated number of people with HTN has risen significantly from 54.6 million in 1990 to 130.2 million in 2010. It is predicted to rise to 216.8 million by the year 2030 which is a 66% rise from the year 2010 [9]. Further studies have shown that of those with HTN, 73% were unrecognized; only 18% received treatment and 7% had a controlled blood pressure measurement [12]. In Ghana, the prevalence of hypertension in adults is reported to range from 19 to 48% across rural and urban communities [13,14], making it the most predominant outpatient condition in the country. More worrying is the fact that about two-thirds of Ghanaian adults with hypertension are unrecognized/unaware of their status and so are unable to take appropriate measures to manage it [15].

Increasing evidence from literature suggests that excess weight gain/obesity is significantly associated with the development of hypertension [16,17]. The use of population-based studies to investigate the link between obesity and hypertension have significant benefits because these findings contribute to the knowledge on the burden of these potentially modifiable factors that can be addressed through public health intervention programs. These studies also help assess the strength of this relationship especially recognizing the influence of regional, ethnic, and geographic diversity [18].

Anthropometric indices have long been used in the characterization of adiposity and obesity as it’s an easy, economical and effective method [19]. Dual-energy X-ray absorptiometry, although considered the gold standard in accurately measuring body composition, may not be available in practice in low-resource countries like Ghana due to its high cost, need for expertise and long turnaround time [20,21]. Traditional anthropometric indices such as body mass index (BMI) waist to hip ratio (WHR), waist circumference (WC) hip circumference (HC), waist to height ratio (WHtR) and conicity index (CI) are commonly used indicators of obesity, as they are comparatively easy and cheap to measure despite their limitations [19,22]. Several other relatively recent and useful anthropometric indices such as Abdominal volume index (AVI), a body shape index (ABSI) and body roundness index (BRI) have been utilized as effective measures of obesity [23,24]. Few studies analyzing the validity and comparison of the old and new indices in predicting HTN have been undertaken elsewhere [18,25,26]. Unsurprisingly, there was variability in the choice of anthropometric indices which demonstrates outcome specific to ethnic and geographic variability. Nonetheless, no study has comprehensively assessed the performance of the relative newly added anthropometric indices and its’ comparability with the traditional indices of obesity in predicting unrecognized hypertension in the Ghanaian adult population. This affirms the need for such study in Ghana. Our study also aimed at the estimation of cut-off points of anthropometric indices which will provide information on population at risk of developing hypertension to undergo formal estimation of their risk. The study will also contribute to knowledge in healthcare facilities and a new dimension could be taken to include the new anthropometric indices in hypertension risk assessment.

## Materials and methods

### Ethics statement

The study was approved by the Committee for Human Research and Publication Ethics (CHRPE), of the School of Medical Sciences (SMS), Kwame Nkrumah University of Science and Technology, Kumasi (CHRPE/AP/474/22). Participation was fully voluntary and written informed consent was obtained from each study participant.

### Study design/study setting

This community based cross-sectional study was conducted between January to August 2022 in the Ablekuma North municipal district. Ablekuma North municipal district is one of the twenty-nine districts in the Greater Accra region, Ghana, located in the Southwestern part of Accra [27]. The Municipal is divided into seven (7) Electoral Areas namely, Odorkor, Darkuman West, Darkuman East, Awoshie, Otaten, Sakaman and Kwashieman. The municipality is advantageously placed, spanning the commercial, industrial, and residential regions of Accra.

### Study population and participants selection

A total of 1,288 study participants were selected based on a multistage sampling approach across the seven (7) electoral areas of Ablekuma North municipality. Adults aged 18–80 years who had no complaint of any illness or known by any disease at the time of the study also known as–apparently healthy adults were recruited into the study. Adults who were either known hypertensive or had hypertensive related chronic illness, individuals with history of hypertension or on anti-hypertension medication were not included in the study. The study did not also include pregnant women, adults with history of chronic illness, diabetes, cardiovascular diseases and renal disease. In the first stage of sampling, four electoral areas were selected using simple random sampling technique, and based on the proportion of size of the electoral areas, we employed a systematic random sampling technique to select households from the electoral areas in the second stage. Then we randomly selected one adult aged 18 years and above and meeting the inclusion criteria from each household using the lottery method.

#### Sample size estimation

Using a single population formula by considering: the prevalence of unrecognized hypertension in a previous study conducted in Ghana (P = 0.387) [28]. Margin of error (D = 0.05), level of significance (α = 0.05), Z α /2 at 95% CI = 1.96 and 10% contingency rate using the Cochrane formula [29],

n = $\frac{Z^{2}P(1-P)}{D^{2}}$, a sample size of 365 was obtained. To increase statistical power a total of 1,288 adults were included in the study.

### Data collection

#### Study questionnaire

Each participant had a structured based interview which was conducted privately and in person. The questionnaire was sectioned into four (4) parts. Section A requested information on the socio-demographic characteristics such as age, gender, marital status, occupation and level of education. Section B required information on lifestyle activities which included alcohol intake, smoking and the level of physical activity. Section C requested details on dietary patterns, mainly fruit and vegetables intake. Information on family history and the current medical history of participants was requested in the last section, Section D. The questionnaire was pre-tested for reliability in a pilot study which yielded a Cronbach alpha value of 0.895.

#### Blood pressure measurement

A registered Nurse used an automatic validated device (Omron HEM711DLX, UK) to measure the blood pressure of all participants. The measurement was done on the upper left arm while the subject was seated with their legs uncrossed, their arm supported at the height of the heart, and the arm wrapped in a cuff that was appropriate for their arm size. Blood pressure was measured after each participant had rested for at least 10 mins. Measurements were repeated twice at 5 mins interval and the average systolic blood pressure and diastolic blood pressure were recorded. Unrecognized hypertension was defined as systolic pressure levels ≥140 mmHg and/or diastolic levels ≥90 mmHg according to the 2018 European Society of Cardiology/European Society of Hypertension Guidelines [30]. The diagnosis of BP was made by a medical practitioner.

#### Anthropometric assessment

Anthropometric measurements constituted height, weight, hip and waist circumference. The subjects’ heights were measured by placing their heels together, leaning their heads on a wall-mounted ruler, and standing up straight in bare feet. Weight was measured using a digital scale (Etekcity EB930H), with participants barefooted and wearing light indoor clothes. WC was measured using a Gulick II spring-loaded measuring tape (Gay Mills, WI) placed horizontally, midway between the lowest costal margin and the anterior superior iliac crest, with participants standing. The reading was obtained after gentle expiration. HC was measured using a measuring tape at the level parallel to the floor, at the largest circumference of the buttock with both hands open widely.

Anthropometric indices were calculated as follows [31], [32], [23], [24];

$$•\;BMI=\frac{weight\;(kg)}{{height\;(m)}^{2}}$$

$$•\;WHR=\frac{WC\;(cm)}{HC\;(cm)}$$

$$•\;WHtR=\frac{WC\;(cm)}{Height\;(cm)}$$

$$•\;CI=\frac{WC\;(m)}{0.109\times \sqrt{\frac{weight\;(kg)}{height\;(m)}}}.$$

$$•\;AVI=\frac{{2WC\;(cm)}^{2}+0.7{\left(WC-HC)\;(cm\right)}^{2}}{1000}$$

$$•\;ABSI=\frac{WC\;(m)}{\left[{BMI}^{\frac{2}{3}}\times {height\;(m)}^{\frac{1}{2}}\right]}$$

$$•\;BRI=364.2-365.5\times \{1-[\frac{\frac{WC}{2\pi }}{{\;(0.5\times height)}^{2}}]{\}}^{0.5}$$

### Statistical analysis

All data was documented in Microsoft Excel 2016, then entered electronically and analyzed using IBM SPSS version 26.0 software and GraphPad Prism version 8.0. The data distribution was tested for normality using the Kolmogorov–Smirnov test. Continuous variables were presented as mean ± standard deviation or median (interquartile range) where appropriate while categorical variables were presented as frequencies and percentages. Comparisons across socio-demographic characteristics such as age groups, sex, educational level, marital status and between hypertensives and non-hypertensives participants were performed using chi-square test. Student’s t-test or Mann–Whitney U test was used for comparing continuous variables and hypertension status where applicable. All anthropometric indices were categorized by quartiles except BMI which used the standard World Health Organization (WHO) categories. Univariate logistic regression analysis followed by the multivariate logistic regression model was performed to determine the association between these categories and HTN after adjusting for age, marital status, alcohol intake and level of exercise. The performance of the anthropometric measures as potential predictors of HTN was compared using the receiver operating characteristic curve (ROC) analysis. The optimal cut-off points for WHR, WHtR, BMI, ABSI, AVI, BRI and CI were determined by the best balance of sensitivity and specificity. Statistical significance was set at *p*<0.05.

## Results

### Socio-demographic characteristics of study population

**Table 1** shows the socio-demographic characteristics of the study population. A total of 1288 adult participants were recruited for the study. The majority of the participants were in the age categories 18–34 (36.5%). Age categories was significantly associated with unrecognized hypertension (HTN) status of participant (*p* < 0.001). Majority of the participants were females (53.3%) with a male to female ratio of 1:1.1. Gender was significantly associated with unrecognized HTN (*p* = 0.026). The highest proportion of the participants was married (55%) and marital status of participants was found to be significantly associated with unrecognized HTN (*p* <0.001). Also, level of education (*p* = 0.027), occupation (*p* = 0.004*)*, alcohol intake status (*p* < 0.001) and exercise level (*p* < 0.001) were all significantly associated with unrecognized HTN.

**Table 1:** Socio-demographic characteristics of the study population.

		Unrecognized HTN		
Variable	Total (n = 1288)	No (n = 1051)	Yes (n = 237)	*p*-value
**Age categories (years)**				**<0.001**
18–34	470(36.5)	423(40.2)	47(19.8)	
35–44	333(25.9)	276(26.3)	57(24.1)	
45–54	300(23.3)	219(20.8)	81(34.2)	
55–79	185(14.4)	133(12.7)	52(21.9)	
**Gender**				**0.026**
Male	601(46.7)	506(48.1)	95(40.1)	
Female	687(53.3)	545(51.9)	142(59.9)	
**Marital status**				
Married	709(55.0)	567(53.9)	142(59.9)	**<0.001**
Single	495(38.4)	436(41.5)	59(24.9)	
Divorced	66(5.1)	38(3.6)	28(11.8)	
Widowed	18(1.4)	10(1.0)	8(3.4)	
**Ethnicity**				0.254
Akan	1060(82.3)	874(83.2)	186(78.5)	
Ewe	51(4.0)	37(3.5)	14(5.9)	
Ga	150(11.6)	119(11.3)	31(13.1)	
Mole Dagbani	27(2.1)	21(2.0)	6(2.5)	
**Level of education**				**0.027**
No education	30(2.3)	26(2.5)	4(1.7)	
Basic	378(29.3)	297(28.3)	81(34.2)	
Secondary	543(42.2)	436(41.5)	107(45.1)	
Tertiary	337(26.2)	292(27.8)	45(19.0)	
**Occupation**				**0.004**
Unemployed	233(18.1)	206(19.6)	27(11.4)	
Formal	447(34.7)	367(35.0)	80(33.8)	
Informal	608(47.2)	478(45.5)	130(54.9)	
**Alcohol intake**				**<0.001**
Occasionally	207(16.1)	172(16.4)	35(14.8)	
Weekly	51(4.0)	30(2.9)	21(8.9)	
Daily	18(1.4)	8(0.8)	10(4.2)	
Never	1012(78.6)	841(80.0)	171(72.2)	
**Fruit intake**				0.149
Two or more times a week	590(45.8)	471(44.8)	119(50.2)	
Once a week/Never	698(54.2)	580(55.2)	118(49.8)	
**Vegetable intake**				0.494
Two or more times a week	994(77.2)	815(77.5)	179(75.5)	
Once a week/Never	294(22.8)	236(22.5)	58(24.5)	
**Regular physical exercise**				**<0.001**
Two or more times a week	417(32.4)	374(35.6)	43(18.1)	
Once a week/Never	871(67.6)	677(64.4)	194(81.9)	
**Family medical history**				0.577
Hypertension	31(2.4)	25(2.4)	6(2.5)	
Stroke	60(4.7)	45(4.3)	15(6.3)	
Diabetes	45(3.5)	36(3.4)	9(3.8)	
None	1152(89.4)	945(89.9)	207(87.3)	

Data is presented as Chi-square or Fisher’s test. P < 0.05 was considered significant.

### Prevalence of unrecognized hypertension among the study population

**Fig 1:**
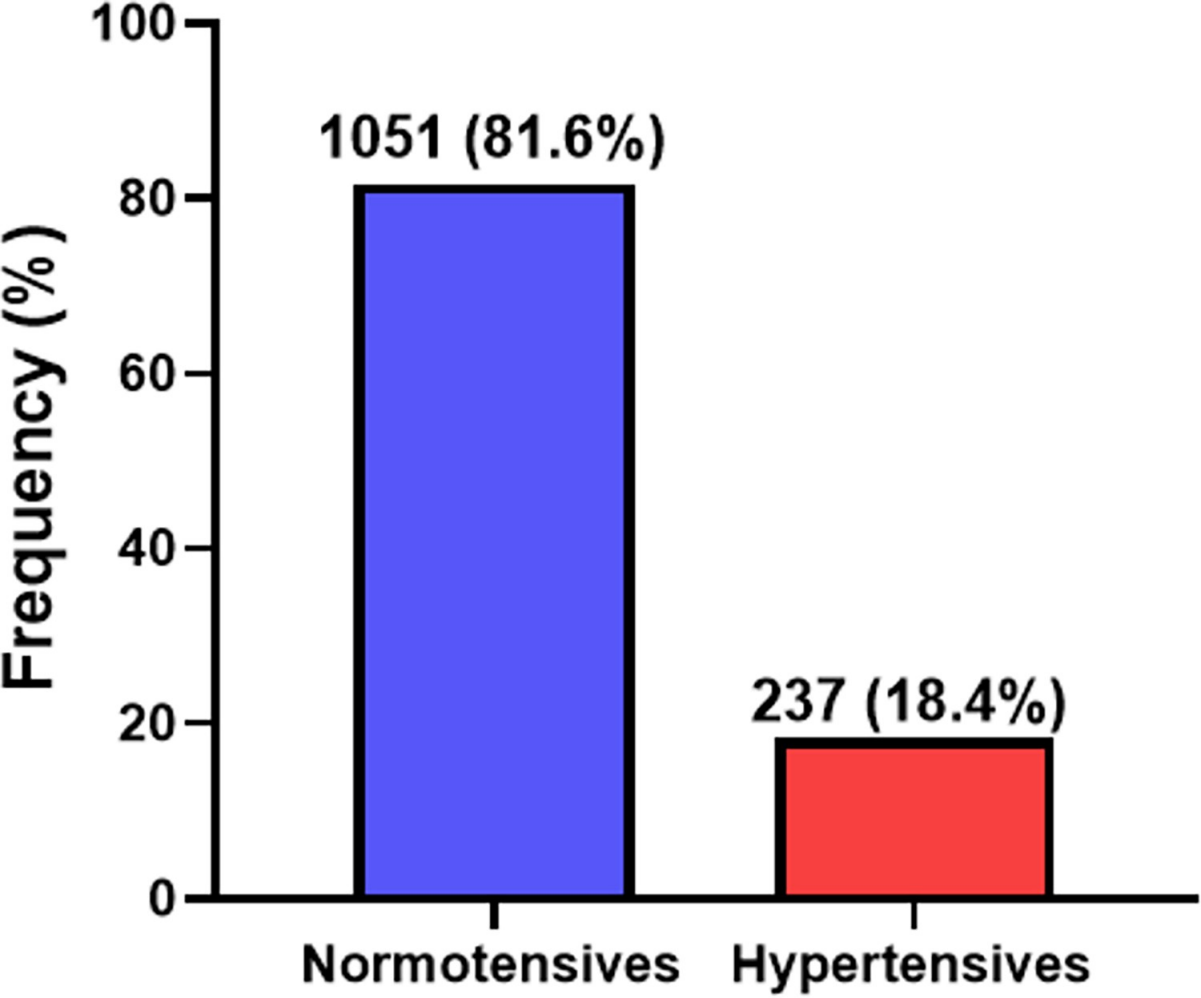
Prevalence of unrecognized hypertension among the general adult population.

**Fig 2:**
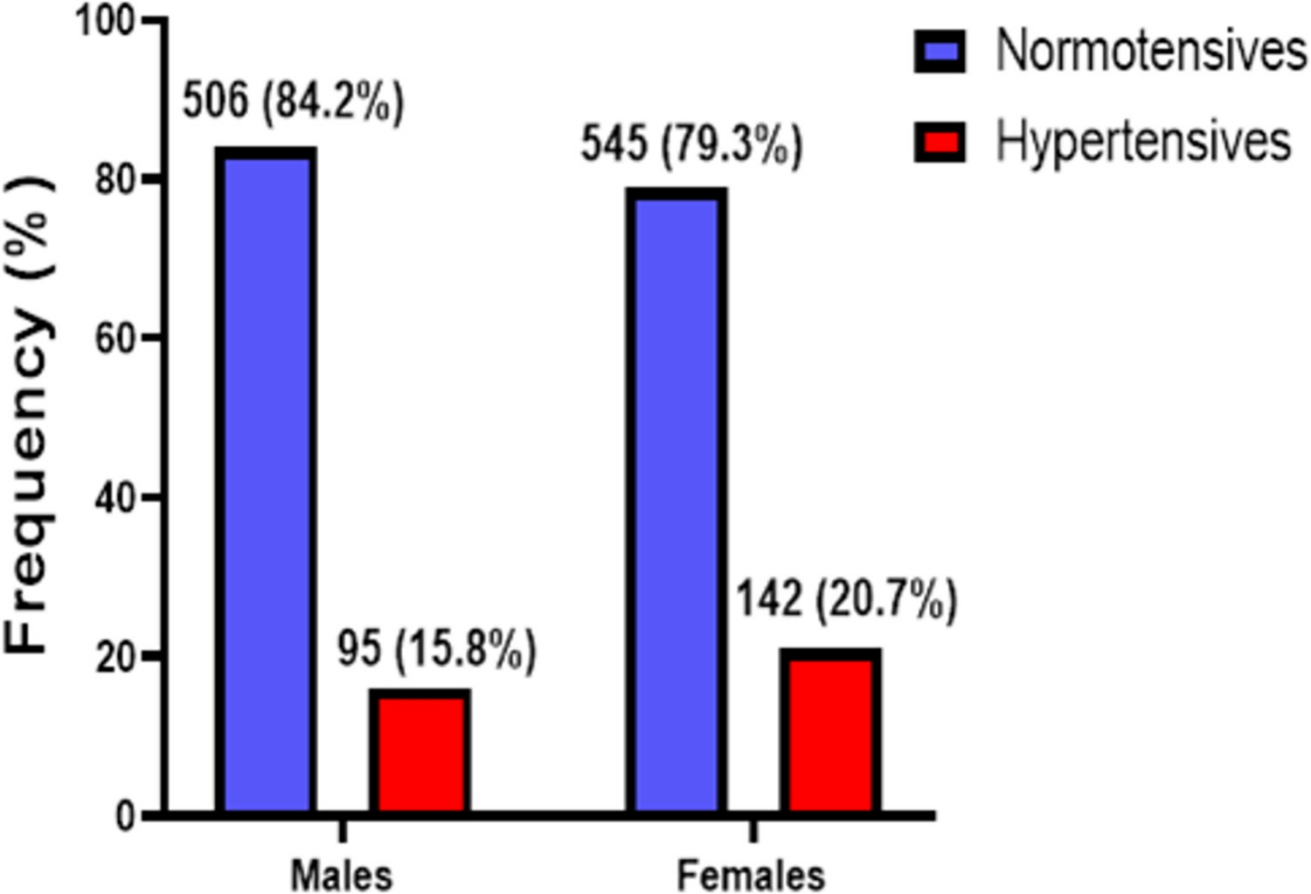
Prevalence of unrecognized hypertension among male and female populations.

The overall prevalence of unrecognized hypertension among the study population was 18.4% (237/1288) as shown in **Fig 1.** Also, as shown in **Fig 2**, the prevalence of unrecognized hypertension was high in females than male participants (20.7% vs 15.8%) **Fig 2**.

### Anthropometric variables of the study population

**Table 2** shows the baseline characteristics and anthropometric variables of the general study population. The median age of the total participants was 39 years. Participants with hypertension had significantly higher age median levels compared to normotensives (49 years versus 37 years, *p* <0.001).

**Table 2:** Anthropometric variables of the study population.

		Unrecognized HTN		
Variable	Total (n = 1288)	No (n = 1051)	Yes (n = 237)	*P* value
**Age**	39.00 (28.00–49.00)	37.00 (26.00–46.00)	49.00 (44.00–56.00)	**<0.001**
**SBP (mmHg)**	117.00 (106.50–129.00)	112.50 (104.50–121.00)	145.00 (141.50–151.00)	**<0.001**
**DBP (mmHg)**	70.00 (64.50–83.00)	67.50 (62.00–75.50)	91.50 (87.50–97.00)	**<0.001**
**Height (cm)**	166.00 (160.00–170.00)	166.40 (160.00–171.00)	165.50 (158.80–168.00)	**0.032**
**Weight (kg)**	70.30 (63.10–79.00)	69.90 (62.38–78.08)	72.30 (68.90–83.00)	**<0.001**
**WC (cm)**	85.70 ± 10.70	84.16 ± 9.92	92.52 ± 8.77	**<0.001**
**HC (cm)**	98.30 (92.25–106.40)	97.70 (91.65–104.80)	102.50 (97.60–109.10)	**<0.001**
**BMI (kg/m2)**	25.03 (23.24–29.09)	24.75 (22.97–28.51)	28.21 (24.72–30.97)	**<0.001**
**WHR**	0.86 (0.82–0.90)	0.85 (0.81–0.90)	0.88 (0.84–0.93)	**<0.001**
**WHtR**	0.52 ± 0.07	0.51 ± 0.07	0.57 ± 0.06	**<0.001**
**BRI**	3.75 (2.72–4.74)	3.59 (2.52–4.54)	4.68 (3.78–5.44)	**<0.001**
**ABSI (m11/6kg-2/3)**	0.076 ± 0.006	0.076 ± 0.006	0.078 ± 0.006	**0.020**
**AVI (cm2)**	15.08 ± 3.51	14.55 ± 3.34	17.41 ± 3.30	**<0.001**
**CI (m3/2/kg1/2)**	1.21 (1.14–1.27)	1.19 (1.12–1.25)	1.26 (1.20–1.31)	**<0.001**

Non-parametric data are presented as median (interquartile range); compared using Mann-Whitney test. *P* < 0.05 was considered statistically significant. Parametric data are presented as mean ± SD; compared using independent sample t-test. P <0.05 was considered statistically significant. N: Number. IQR: Interquartile range. SD: Standard deviation. SBP: Systolic blood pressure. DBP: Diastolic blood pressure. BMI: Body mass index. CI: Conicity index. ABSI: A body shape index. BRI: Body roundness index. AVI: Abdominal volume index. WC: Waist circumference. HC: Hip circumference. WHR: Waist-to-hip ratio. WHtR: Waist-to-height ratio.

Except for the median height which was significantly high amongst normotensives participants than hypertensives subjects (166.40 cm versus 165.50 cm, *p* = 0.032), all other anthropometric indices including weight, BMI, ABSI, AVI, BRI, CI, WHR and WHtR had significantly higher median or mean levels amongst hypertensives compared to normotensives (*p* < 0.05). Overall, the median systolic blood pressure (SBP) (145.00 vs 112.50) and diastolic blood pressure (DBP) (91.50 vs 67.50) was significantly higher among participants with hypertension compared to normotensives (*p* < 0.001).

### Gender-specific anthropometric variables of the study population

**Table 3** shows the gender specific anthropometric variables of the general study population. The median age of hypertension was significantly higher in hypertensives than normotensives in male participants (47.5 years versus 36 years, *p <* 0.001). A similar trend was seen among female participants (49 years versus 37 years, *p* < 0.001*)*. Among the male participants, the median height was significantly high in normotensives compared to hypertensives. Except for ABSI (*p* = 0.067) and weight (*p =* 0.063) all other anthropometric indices had significantly higher levels amongst hypertensives compared to normotensives (*p* < 0.05). Among the female participants, all anthropometric indices were significantly high in hypertensives (*p* <0.05) except for height (*p* = 0.859) compared to normotensive females. For both male and female, the median systolic blood pressure (SBP) and diastolic blood pressure (DBP) was significantly higher among participants with hypertension compared to normotensives (*p* < 0.001).

**Table 3:** Gender-specific anthropometric variables of the study population.

		Unrecognized HTN		
Variable	Total (n = 1288)	No (n = 1051)	Yes (n = 237)	*P* value
**Males**				
**Age**	38.00 (26.00–48.00)	36.00 (24.00–46.75)	47.50 (39.25–55.75)	**<0.001**
**SBP (mmHg)**	117.00 (107.63–126.50)	113.00 (106.63–122.88)	149.50 (142.63–152.38)	**<0.001**
**DBP (mmHg)**	70.00 (65.13–79.00)	68.00 (64.00–75.50)	92.25 (89.00–98.75)	**<0.001**
**Height (m)**	169.85 (169.63–173.60)	170.00 (167.00–175.13)	168.00 (164.25–170.75)	**0.018**
**Weight (kg)**	71.13 (66.05–79.20)	70.75 (65.43–78.85)	72.55 (68.78–83.75)	0.063
**WC (cm)**	83.52 ± 9.33	82.42 ± 8.85	89.28 ± 11.55	**0.001**
**HC (cm)**	94.70 (90.00–99.00)	94.00 (89.50–98.85)	98.30 (94.25–102.60)	**0.004**
**BMI (kg/m2)**	24.42 (22.69–27.03)	24.30 (22.39–26.55)	25.81 (24.09–29.92)	**0.004**
**WHR**	0.87 (0.83–0.92)	0.87(0.83–0.91)	0.89 (0.86–0.94)	**0.014**
**WHtR**	0.49 ± 0.06	0.48 ± 0.05	0.54 ± 0.07	**<0.001**
**BRI**	3.13 (2.36–3.88)	2.95 (2.31–3.73)	3.78 (3.27–4.97)	**<0.001**
**ABSI (m11/6kg-2/3)**	0.075 ± 0.006	0.075 ± 0.006	0.077 ± 0.007	0.067
**AVI (cm2)**	14.25 ± 3.18	13.85 ± 2.77	16.68 ± 4.37	**0.001**
**CI (m3/2/kg1/2)**	1.16 (1.11–1.24)	1.16 (1.11–1.23)	1.23 (1.15–1.30)	**0.008**
**Females**				
**Age**	41.00 (32.00–49.00)	37.00 (28.00–46.00)	49.00 (44.00–56.00)	**<0.001**
**SBP (mmHg)**	117.00 (104.75–134.50)	111.25 (102.50–120.13)	142.50 (141.00–149.50)	**<0.001**
**DBP (mmHg)**	70.00 (64.00–81.00)	67.00 (61.38–75.13)	90.75 (86.50–96.00)	**<0.001**
**Height (m)**	161.00 (156.20–166.10)	160.95 (156.88–165.70)	161.50 (155.00–167.00)	0.859
**Weight (kg)**	70.00 (61.90–78.63)	68.43 (60.09–78.00)	73.00 (68.90–82.25)	**<0.001**
**WC (cm)**	87.60 ± 10.63	85.85 ± 10.92	93.73 ± 6.62	**<0.001**
**HC (cm)**	103.50 (96.00–109.00)	102.50 (95.00–109.63)	107.00 (99.50–111.50)	**0.011**
**BMI (kg/m2)**	26.45 (24.03–30.06)	25.96 (23.68–29.49)	28.91 (24.86–32.90)	**<0.001**
**WHR**	0.85 (0.81–0.89)	0.84 (0.80–0.88)	0.87 (0.83–0.93)	**<0.001**
**WHtR**	0.55 ± 0.07	0.53 ± 0.07	0.58 ± 0.05	**<0.001**
**BRI**	4.35 (3.52–5.23)	4.10 (3.12–5.02)	4.99 (4.19–6.10)	**<0.001**
**ABSI (m11/6kg-2/3)**	0.077 ± 0.006	0.077 ± 0.006	0.078 ± 0.005	**0.023**
**AVI (cm2)**	15.80 ± 3.62	15.22 ± 3.69	17.82 ± 2.50	**<0.001**
**CI (m3/2/kg1/2)**	1.24 (1.16–1.28)	1.23 (1.15–1.27)	1.27 (1.22–1.31)	**<0.001**

Non-parametric data is presented as median (interquartile range); compared using Mann-Whitney test. P < 0.05 was considered statistically significant. Parametric data is presented as mean ± SD; compared using independent sample t-test. P <0.05 was considered statistically significant. N: Number. IQR: Interquartile range. SD: Standard deviation. SBP: Systolic blood pressure. DBP: Diastolic blood pressure. BMI: Body mass index. CI: Conicity index. ABSI: A body shape index. BRI: Body roundness index. AVI: Abdominal volume index. WC: Waist circumference. HC: Hip circumference. WHR: Waist-to-hip ratio. WHtR: Waist-to-height ratio.

### Sociodemographic characteristics associated with unrecognized hypertension

**Table 4** shows the univariate and multivariate-adjusted odds of socio-demographic characteristics in association with unrecognized HTN among the study population. In the multivariate analysis being in age groups 45–54, 55–79, divorced, weekly and daily intake of alcohol and exercising at most once a week were independently associated with unrecognized hypertension.

**Table 4:** Sociodemographic characteristics associated with unrecognized hypertension.

Variable	cOR (95% CI)	*p*-value	aOR (95% CI)	*p*-value
**Age group(years)**				
18–34 **(ref)**	1.000		1.000	
35–44	1.859 (1.228–2.814)	**0.003**	1.190(0.955–1.483)	0.121
45–54	3.329 (2.490–4.940)	**< 0.001**	2.288(1.325–3.952)	**0.003**
55–79	3.519 (2.266–5.463)	**<0.001**	3.245(1.610–6.542)	**0.001**
**Gender**				
Male **(ref)**	1.000		1.000	
Female	1.388(1.042–1.848)	**0.025**	1.269(0.670–1.809)	0.188
**Marital status**				
Married **(ref)**	1.000		1.000	
Single	0.540(0.389–0.750)	**<0.001**	0.724(0.450–1.165)	0.183
Divorced	2.942(1.746–4.957)	**<0.001**	3.019(1.334–6.900)	**0.008**
Widowed	3.194(1.238–8.241)	**0.023**	2.211(0.663–7.400)	0.198
**Level of education**				
No formal education	0.998(0.333–2.994)	0.986	0.223(0.019–2.612)	0.232
Basic	1.770(1.188–2.636)	**0.005**	1.117(0.769–1.622)	0.561
Secondary	1.592(1.091–2.325)	**0.016**	1.015(0.948–1.086)	0.667
Tertiary **(ref)**	1.000		1.000	
**Occupation**				
Unemployed **(ref)**	1.000		1.000	
Formal	1.663(1.041–2.657)	**0.029**	1.256(0.948–1.742)	0.172
Informal	2.075(1.329–3.240)	**0.002**	0.935(0.790–1.107)	0.433
**Alcohol intake**				
Occasional	1.001(0.672–1.491)	0.997	1.500(0.623–3.611)	0.366
Weekly	3.443(1.925–6.157)	**<0.001**	4.101(1.769–9.505)	**0.001**
Daily	6.148(2.392–15.803)	**<0.001**	5.619(1.262–12.326)	**0.028**
Never **(ref)**	1.000			
**Exercise**				
Two or more times a week **(ref)**	1.000		1.000	
Once a week/Never	2.492(1.750–3.550)	**<0.001**	2.253(1.556–3.655)	**0.001**

cOR, Crude Odd ratio; CI, Confidence interval; aOR Adjusted Odd ratio; Compared using univariate and multivariate logistic regression analysis. *P*-value of < 0.05 was considered statistically significant. 1.000 indicates reference category.

#### Anthropometric indices associated with unrecognized hypertension among male and female participants

The odds of association of the various anthropometric indices with unrecognized HTN in male participants were analyzed using logistic regression as shown in **Table 5.** Multivariate regression showed that the fourth quartile (Q4) of BRI [aOR = 5.19, 95% CI (1.05–25.50), *p* = 0.043] and WHtR [aOR = 5.19, 95% CI (1.05–25.50), *p* = 0.043] were shown to be independent determinant of HTN.

**Table 5:** Anthropometric indices associated with unrecognized hypertension in males.

Variable	cOR (95% CI)	*p*-value	aOR (95%) CL	*p*-value
**ABSI quartiles**				
Q1	Ref (1)			
Q2	0.458 (0.108–1.944)	0.290	-	
Q3	1.194 (0.371–3.841)	0.766	-	
Q4	2.278 (00.813–2.378)	0.114	-	
**AVI quartiles**				
Q1	Ref (1)			
Q2	2.089 (0.492–8.860)	0.318	1.481 (0.332–6.609)	0.607
Q3	3.439 (0.872–13.563)	0.078	2.363 (0.559–10.001)	0.243
Q4	4.017 (1.033–15.624)	**0.045**	2.373 (0.584–9.642)	0.227
**BRI quartiles**				
Q1	Ref (1)			
Q2	1.565 (0.250–9.800)	0.409	1.628 (0.252–10.533)	0.609
Q3	6.286 (1.318–29.988)	**0.021**	4.830 (0.980–23.814)	0.053
Q4	8.000(1.684–37.997)	**0.009**	5.186 (1.054–25.504)	**0.043**
**CI quartiles**				
Q1	Ref (1)			
Q2	1.335 (0.337–5.295)	0.465	1.135 (0.274–4.705)	0.862
Q3	1.913 (0.523–6.992)	0.487	1.054 (0.414–6.071)	0.502
Q4	3.711 (1.107–12.439)	**0.034**	2.497 (0.712–8.817)	0.152
**WHR quartiles**				
Q1	Ref (1)			
Q2	2.182 (0.514–9.225)	0.659	2.403 (0.451–12.793)	0.914
Q3	3.512 (0.891–13.843)	0.129	3.566 (0.695–18.304	0.619
Q4	4.103 (1.055–15.947)	**0.042**	3.308 (0.649–16.865)	0.982
**WHtR quartiles**				
Q1	Ref (1)			
Q2	1.565 (0.250–9.800)	0.409	1.628 (0.252–10.533)	0.609
Q3	6.286 (1.318–29.988)	**0.021**	4.830 (0.980–23.814)	0.053
Q4	8.000(1.684–37.997)	**0.009**	5.186 (1.054–25.504)	**0.043**
**BMI categories**				
Normal	Ref (1)			
Overweight	1.1872 (0.746–4.694)	0.181	1.203 (0.459–3.151)	0.707
Obese	4.773 (1.589–14.331)	**0.005**	3.019 (0.948–9.609)	0.061

cOR: Crude odds ratio. aOR: Adjusted odds ratio. Inf: Infinity. Ref: Reference. Compared using univariate and multivariate logistic regression. p < 0.05 was considered significant. Adjusted for age, marital status, alcohol intake and exercise level of participants. Q1: First quartile. Q2: Second quartile. Q3: Third quartile. Q4: Fourth quartile. ABSI: A body shape index. BRI: Body roundness index. BMI: Body mass index. CI: Conicity index. WHtR: Waist-to-height ratio. WHR: Waist-to-hip ratio.

Among female participants, Q3 and Q4 of AVI, BRI and WHtR were independent predictors of unrecognized HTN in multivariate logistic regression as shown in **Table 6 {**AVI quartiles; Q3 [aOR = 7.96, 95% CI (1.51–42.52), *p* = 0.015] and Q4 [aOR = 9.87 95% CI (1.92–53.31), *p* = 0.007], BRI and WHtR quartiles; Q3 [aOR = 6.07, 95% CI (1.05–34.94), *p* = 0.044] and Q4 [aOR = 9.76, 95% CI (1.74–54.96), *p* = 0.010]}.

**Table 6:** Anthropometric indices associated with unrecognized hypertension in females.

Variable	cOR (95% CI)	*p*-value	aOR (95%) CL	*p*-value
**Females**				
**ABSI quartiles**				
Q1	Ref (1)			
Q2	1.026 (0.384–2.739)	0.960	0.898 (0.299–2.693)	0.848
Q3	1.736 (0.683–4.414)	0.246	0.954 (0.326–2.799)	0.932
Q4	2.743 (1.103–6.821)	**0.030**	1.286 (0.434–3.814)	0.650
**AVI quartiles**				
Q1	Ref (1)			
Q2	5.729 (1.196–27.449)	**0.029**	3.944 (0.690–22.553)	0.113
Q3	11.687 (2.554–53.476)	**0.002**	7.957 (1.510–42.515)	**0.015**
Q4	17.286 (3.825–78.114)	**<0.001**	9.874 (1.921–53.310)	**0.007**
**BRI quartiles**				
Q1	Ref (1)			
Q2	8.750 (1.888–40.559	**0.006**	5.179 (0.946–30.768)	0.070
Q3	9.821 (2.129–45.316)	**0.003**	6.065 (1.053–34.936)	**0.044**
Q4	14.865 (3.277–67.431)	**<0.001**	9.764 (1.735–54.962)	**0.010**
**CI quartiles**				
Q1	Ref (1)			
Q2	2.713 (0.889–8.283)	0.122	3.137 (0.906–10.857)	0.071
Q3	3.714 (1.248–11.056)	**0.018**	1.854 (0.527–6.616)	0.336
Q4	5.200 (1.783–15.164)	**0.003**	1.984 (0.290–1.984)	0.290
**WHR quartiles**				
Q1	Ref (1)			
Q2	2.655 (0.089–8.283)	0.086	1.471 (0.420–5.158)	0.546
Q3	3.805 (1.277–11.337)	**0.016**	2.809 (0.835–9.450)	0.095
Q4	5.200 (1.783–15.164)	**0.003**	2.032 (0.609–6.777)	0.249
**WHtR quartiles**				
Q1	Ref (1)			
Q2	8.750 (1.888–40.559	**0.006**	5.179 (0.946–30.768)	0.067
Q3	9.821 (2.129 (45.316)	**0.003**	6.065 (1.053–34.936)	**0.044**
Q4	14.865 (3.277–67.431)	**<0.001**	9.764 (1.735–54.962)	**0.010**
**BMI categories**				
Normal	Ref (1)			
Overweight	1.605 (0.733–3.516)	0.237	1.356 (0.539–3.414)	0.517
Obese	3.012 (1.371–6.617)	**0.006**	2.477 (0.976–6.284)	0.056

cOR: Crude odds ratio. aOR: Adjusted odds ratio. Inf: Infinity. Ref: Reference. Compared using univariate and multivariate logistic regression. p < 0.05 was considered significant. Adjusted for age, marital status, alcohol intake status and exercise level of participants. Q1: First quartile. Q2: Second quartile. Q3: Third quartile. Q4: Fourth quartile. ABSI: A body shape index. BRI: Body roundness index. BMI: Body mass index. CI: Conicity index. WHtR: Waist-to-height ratio. WHR: Waist-to-hip ratio.

#### Predictive potential of the various anthropometric indices for unrecognized hypertension in male and female participants

**Fig 3:**
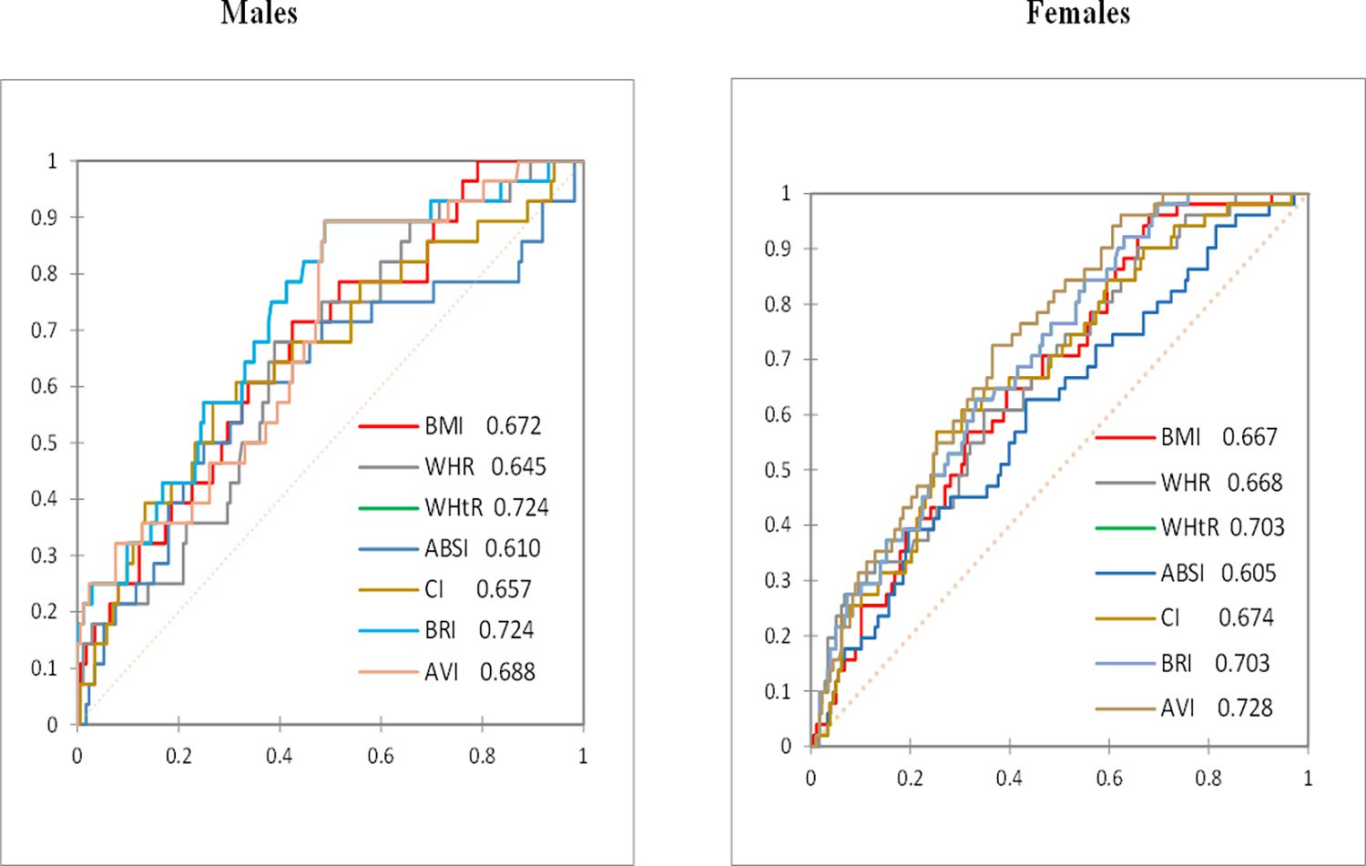
ROC curves of anthropometric indices for males and females.

**Table 7** demonstrates the predictive performance of the various anthropometric indicators based on ROC curve analysis. Among male participants, the highest AUC was recorded for BRI and WHtR (0.724; 95% CI: 0.623–0.824) and AVI (0.688; 95% CI: 0.585–0.791). BMI recorded an AUC of 0.672 (95% CI: 0.567–0.777). The optimal WHtR, BRI and BMI cut-offs for identifying males with HTN were 0.480 (sensitivity 0.89 and specificity 0.51), 2.982 (sensitivity 0.89 and specificity 0.51), and 24.633 (sensitivity 0.71 and specificity 0.58). respectively. AVI (0.728; 95% CI: 0.658–0.799), WHtR (0.703; 95% CI: 0.628–0.777) and BRI (0.703; 95% CI: 0.628–0.777) recorded the highest AUC values among female subjects. However, ABSI recorded the lowest AUCs for both male and female participants (0.610 and 0.605 respectively). **Fig 3** compares the ROC curves of the various anthropometric indices for males and females respective.

**Table 7:** Anthropometric indices for predicting unrecognized hypertension.

	Variable	AUC (95% CI)	Cut off	Sensitivity	Specificity	PPV	NPV
**Males**	BMI	0.672 (0.567–0.777)	24.633	0.714	0.576	0.215	0.925
	WHR	0.645 (0.540–0.750)	0.882	0.679	0.610	0.221	0.921
	**WHtR**	**0.724 (0.623–0.824)**	**0.480**	**0.893**	**0.512**	**0.229**	**0.967**
	**BRI**	**0.724 (0.623–0.824)**	**2.982**	**0.893**	**0.512**	**0.229**	**0.967**
	ABSI	0.610 (0.483–0.738)	0.077	0.607	0.674	0.223	0.913
	AVI	0.688 (0.585–0.791)	13.610	0.893	0.512	0.229	0.967
	CI	0.657 (0.559–0.789)	1.216	0.571	0.733	0.258	0.913
**Females**	BMI	0.667 (0.588–0.744)	27.214	0.647	0.601	0.317	0.856
	WHR	0.668 (0.587–0.749)	0.862	0.608	0.652	0.333	0.853
	**WHtR**	**0.703 (0.628–0.777)**	**0.566**	**0.627**	**0.669**	**0.352**	**0.862**
	**BRI**	**0.703 (0.628–0.777)**	**4.679**	**0.627**	**0.669**	**0.352**	**0.862**
	ABSI	0.605 (0.517–0.693)	0.081	0.401	0.803	0.364	0.822
	**AVI**	**0.728 (0.658–0.799)**	**16.360**	**0.725**	**0.635**	**0.363**	**0.890**
	CI	0.674 (0.593–0.755)	1.269	0.569	0.747	0.392	0.858

ROC determined cut-off, sensitivity and specificity of each anthropometric index for predicting unrecognized hypertension, ROC receiver operating curve, AUC area under the curve, PPV positive predictive value, NPV negative predictive value.

### Discussion

Evidence for the association between hypertension and obesity abounds and for the first time, this study evaluated the prevalence of unrecognized hypertension and its association with lifestyle factors and obesity indices among the adult population in the Ablekuma North Municipality.

The prevalence of unrecognized HTN in the current study was 18.4% which was comparable to a study in the Urban communities of Southwest Ethiopia [33] but higher compared to a study done in Addis Ababa, Ethiopia [34]. It was however, lower compared to studies among Bus Drivers [28], traders at regional markets in Nigeria [35] and a systematic review in sub-Saharan Africa [12]. Different sociodemographic traits of research participants could be to blame for the observed reduced risk in the current study. For instance, the studies in Ghana among bus drivers, Nigeria among traders at regional markets, and the systematic review in sub-Sahara were conducted on populations with greater risk to HTN, in contrast to the current study, which was conducted on the general adult population. In our study, the prevalence of unrecognized hypertension was high in females (22.3%) as compared to males (14%). This finding is consistent with studies conducted in the Kumasi metropolis in Ghana [36] but unusual as compared to most studies [12,33]. This higher risk of HTN in females may be due to the advanced median ages of the female study participants. Majority of the hypertensive females in this study were in the age region of menopausal transition (44–55years). This period is usually associated with a physiological reduction in ovarian hormones and leads to progressive increase in blood pressure [37,38]. The low physical activity and high overweight/obesity rate in our female participants also play a role.

Our study reported that being in the age groups of 45–54 and ≥ 55 were associated with unrecognized HTN. This can be explained by the loss in vascular compliance as a result of artery hardening and stiffness with increasing age, which contributes to high prevalence of HTN in older groups [39].

In this study, marital status of participants was significantly associated with unrecognized HTN status. Being divorced was associated with a significant 4-folds increase of having HTN compared to being married even after adjustment for possible confounders. Getachew and colleagues reported a similar finding in a cross-sectional study conducted among Ethiopian adults in Addis Ababa [34]. The driving factor is, however, not well understood. These findings may be possibly due to psychological constructs of divorce-related emotional intrusion-hyperarousal as well as lack of social support [40].

Furthermore, weekly and daily intake of alcohol were associated with HTN. Owiredu et al. reported a similar finding [41]. The exact mechanism that alcohol affects blood pressure is still unknown. Several mechanisms have been put forth, including imbalances in the central nervous system, baroreceptor dysfunction, stimulation of the renin-angiotensin-aldosterone system, and elevated vascular reactivity as a result of higher intracellular calcium levels [42].

Also, this study found a significant association between the exercise level of participants and unrecognized HTN. Never exercising or exercising once a week was associated with increased odds of having HTN. This finding is consistent with several cross-sectional studies [41,43] who reported that not exercising routinely was associated with increasing blood pressure Low physical activity is a driving factor for obesity related health complications.

Controversies still exist as to which anthropometric index of obesity is highly associated and can best predict the occurrence of hypertension among the general adult population. Advanced research has led to the development of new and affordable indices. BMI is the most commonly used obesity measure and has been associated with HTN. However, in this present study, BMI showed a poor predictability performance for unrecognized HTN compared to the best performing indices of central obesity (WHtR, BRI, AVI). In the logistic regression model, BMI failed to independently predict unrecognized hypertension. Consequently, BMI showed lower AUC values of 0.672 for the males versus 0.667 for females in the ROC analysis compared to WHtR, BRI, and AVI, which yielded better discriminatory power (AUC ≥7.0) in predicting HTN. This is in keeping with several previous studies that have reported BMI as an inferior predictor of HTN compared to central adiposities indices [18,44,45]. A plausible reason for this observation is that BMI is limited in its ability to differentiate between fat and muscle mass, as well as between various fat compartments such as visceral adipose tissue and subcutaneous tissue [46]. However, other studies have reported that BMI has a similar predictability performance as other measures of central adiposity [47,48]. This disparity may be due to variations in the sample size, differences in the median age group of participants and geographical location.

ABSI was proposed to predict the risk of pathologies that cannot be readily identified by BMI [23]. Nevertheless, in our study, ABSI showed the poorest association with hypertension across gender as evidenced by an AUC of 0.610 in males and 0.605 in females. Previous studies have also indicated similar findings; ABSI is a very weak predictor of hypertension [18,26]. A probable reason for ABSI’s failure to predict hypertension is that it was originally designed as a mortality risk predictor in a cohort study. In contrast, we determined it predictive potential for hypertension in a cross-sectional study and this may be the primary reason ABSI showed poor association with hypertension.

The association of AVI with unrecognized hypertension was also examined in our study. AVI stood out as an independent predictor of unrecognized HTN after possible confounders were controlled in the multivariate logistic model in females but not males. An increase in HC causes an increase in AVI when WC < HC in the AVI formula. Since females have a larger HC than males do, an increase in HC values typically results in an increase in AVI values, which explains the differences in the two genders’ predictive skills [49].

WHtR and BRI showed similar predictability performance for unrecognized HTN in our study. WHtR and BRI were associated with HTN across genders. It consequently showed better discrimination for HTN than BMI for both male (0.724 versus 0.672) and female participants (0.703 versus 0.667). Our findings concur with several other studies [18,26,36]. One major factor for WHtR and BRI to strongly predict HTN is their ability to rightfully measure visceral/central fat deposits which is a proposed contributor to the incidence of HTN. The exact pathogenesis of hypertension associated with central obesity remains unclear. However, the systematic activity of visceral fat deposits provides a plausible cause for the strong association between central obesity and HTN. Visceral fat deposits are noted to be metabolic active and its accumulation stimulate the production of a variety of pro-inflammatory cytokines such as imterlukin-6 (IL-6) and tumor necrosis factor-alpha (TNF-a), as well as adipokines such as leptin. These molecules initiate a series of events characterized by inflammation, arterial stiffness and impaired vascular function. In addition, these molecules enhance insulin resistance which can lead to the concurrent stimulation of the sympathetic nervous system, all of which contribute to the onset of hypertension [50,51].

Also, this present study determined cut-off values that may be used to gauge normalcy and evaluate the risk of HTN in the Ghanaian setting. The WHtR cut-off levels in this study for males (0.48) and females (0.57) were slightly lower and slightly higher respectively, when compared to the generic cut-off values proposed by WHO (0.5 for adults). The cut-off value we determined for BMI in females (27.2) was higher than the established standard cut-off values by WHO (24.9) but similar cut-off for males (24.63). This differences between the observed values from the standard and recommended values by WHO is not surprising because geographical and ethnic factors can influence the optimal cut-off values of these obesity measures that predict the risk for HTN [18,48]. Moreover, our females generally had higher cut-off values for these obesity indices than males which is different to the WHO established values. However, this finding is consistent with earlier studies among Ghanaian, Nigerian and Iranian adults [18,36,52]. Ghanaian and other African women typically view higher weights as desirable due to the traditional associations between such features and beauty and wealth [36]. Lower physical activity in females is also a factor.

This study had some notable significance as it employed various obesity indices, which made it possible to investigate and ascertain the most effective indices for predicting HTN and their potential usefulness in screening programs and individual assessment of one’s health. Also, this study employed reliable statistical techniques which enabled us indicate the irregularities in pre-established optimal thresholds from our determined values. Despite these findings, it is important to recognize some of the limitation of this study. First, because the study was cross-sectional and the disease’s origin was not identified, conclusions concerning a cause-and-effect relationship cannot be drawn. Hence, a longitudinal investigation is necessary. Also, this study did not consider prehypertension which is also known to increase the risk of cardiovascular disease. Future comparative studies should take this category of blood pressure into consideration. Lastly, there is a possibility of discrepancies or errors between different observers when measuring waist circumference and hip circumference. However, anthropometric indices were measured using a defined methodology by well-trained data personnel, so errors were probably at a minimum.

### Conclusion

There is a high prevalence of unrecognized hypertension among perceived healthy Ghanaian adults. The prevalence of unrecognized hypertension is associated with older age, divorce, alcoholic beverage intake, physical inactivity, and obesity. We emphasize that BMI may not be the most effective method for predicting the risk of hypertension and its associated pathologies regardless of its common use in clinical practice. However, we propose that central obesity indices (WHtR, BRI and AVI) are best associated with the risk of HTN and we recommend their frequent inclusion in screening programs (both self and mass) and clinical settings. For simplicity and cost-effective measures WHtR may be preferred and in the near future, assessment of BRI and AVI (especially in females) may be available in a user-friendly software platform that could provide information on individual risk to HTN. Health professionals are needed to create awareness of these risk factors through educational and screening programs along with individual self-check for blood pressure, lifestyle modifications and weight management to prevent the onset of HTN.
